# Improvement of Biocontrol Efficiency of *Hanseniaspora thailandica* Induced by Alginate Oligosaccharide Against Banana Anthracnose Caused by *Colletotrichum musae*

**DOI:** 10.3390/jof11120824

**Published:** 2025-11-21

**Authors:** Yinfeng Wu, Xiaojie Chen, Xiaoting Lai, Xiaomin Ren, Jianqu Hong, Fen Yan

**Affiliations:** College of Biological Science and Engineering, Fuzhou University, Fuzhou 350108, China; wyf2224727915@163.com (Y.W.); 13164320875@163.com (X.C.); lxt17870408560@163.com (X.L.); rxm15877595435@163.com (X.R.); hjq2437644646@163.com (J.H.)

**Keywords:** Alginate oligosaccharide, Banana fruit, Biocontrol efficacy, *Colletotrichum musae*, *Hanseniaspora thailandica*, Transcriptome

## Abstract

Banana anthracnose, caused by *Colletotrichum musae*, causes substantial postharvest losses worldwide, yet effective biocontrol remains limited. Although *Hanseniaspora thailandica* shows potential, its direct application provides inadequate control. This study demonstrates that alginate oligosaccharide (AOS) potently enhances the biocontrol efficacy of *H. thailandica* Lg 3 against this disease. Through in vivo fruit assays, *H. thailandica* Lg 3 that was cultured in AOS-supplemented medium significantly elevated key defense enzymes, specifically ployphenol oxidase (PPO), peroxidase (POD), chitinase (CHI) and β-1,3-glucanase (GLU) in bananas, and enhanced yeast biofilm formation. This led to higher yeast populations in banana wounds and effective suppression of *C. musae* expansion. Furthermore, AOS boosted the activity of critical antioxidant enzymes (CAT, SOD, GPX) in *H. thailandica* Lg 3 under in vivo conditions. RNA-seq analysis of *H. thailandica* Lg 3 revealed corresponding alterations in glutathione metabolism and oxidative phosphorylation pathways following the addition of 10 mg/L AOS to the medium. The 10 mg/L AOS concentration proved most effective, robustly enhancing biocontrol efficacy. Our findings identify AOS-induced *H. thailandica* Lg 3 as a practical, ready-to-test biocontrol strategy that could be developed into a commercial formulation to reduce chemical fungicide dependence in postharvest banana protection.

## 1. Introduction

Bananas are beloved by consumers for their flavor and nutrition; however, they are prone to pathogen invasion during post-harvest transportation and storage, leading to a decrease in quality [1]. During banana storage, anthracnose symptoms emerge as the fruit ripens, initially appearing as brown or black lesions. As the disease progresses, these lesions expand, become sunken, and produce masses of highly infectious spores. Physical injuries to the peel, such as wounds and scratches incurred during handling and transport, exacerbate disease incidence, severely compromising fruit quality for both export and local markets [2]. In recent years, antagonistic yeasts have gained extensive attention for their ease of cultivation, broad antimicrobial spectrum, and high safety [3]. According to the report, some types of yeast can significantly suppress the occurrence of postharvest diseases in fruits and vegetables through competing for nutrients and space, hyperparasitism, and secreting inhibitory substances [4]. Yet, in practical applications, the antagonistic effect of yeast is significantly affected by the external environment and pathogens, resulting in poor adaptability and sustainability, making it difficult to achieve satisfactory control effectiveness [3]. Accordingly, it is necessary to improve the biocontrol defense ability of biocontrol yeasts. The method of inducing spores by elicitors is simple and effective, which has attracted widespread attention from scholars in recent years [5].

The common agents used to enhance antagonistic yeast performance can be divided into four primary categories: nutritional supplements, antioxidants, pH regulators, and complex polysaccharides [5]. Nutrients, including organic nitrogen, serve as potent enhancers of antagonistic yeasts, significantly improving their performance against plant pathogens [6]. Amino acids, such as arginine, play a dual role by enhancing stress resistance and inducing the biosynthesis of antifungal compounds [7]. A pH regulator, for instance, phytic acid, acts through the modification of the growth microenvironment to enhance yeast viability, concurrently inhibiting the growth of pathogenic fungi [8]. Defensive complex polysaccharides like β-glucan and N-acetylglucosamine lead to prolonged activity by enhancing yeast adhesion to surfaces and stimulating the development of protective biofilms [9,10]. Induced cultivation usually regulates yeast physiology by adding elicitors to basal culture media [11,12].

Alginate oligosaccharide (AOS), a widely available oligosaccharide, has very promising application prospects in the field of biological control [13,14,15]. AOS is a soluble and non-toxic compound that functions as a plant bio-stimulant, which modulates plant development and the innate immune system to enhance disease resistance [13]. These properties contribute to its potent biological effects, including antioxidation, anti-inflammation, immune regulation, and plant growth promotion.

As a result, AOS has been widely applied in various fields such as medicine, food, cosmetics, and agriculture [16]. However, to date, no research has been conducted on the physiological regulation and molecular mechanisms by which AOS enhances the biocontrol efficacy of *Hanseniaspora thailandica* Lg 3. The study has shown that AOS acts as a plant elicitor, regulating plant growth and signal transduction systems to strengthen plant defenses [17]. For instance, postharvest treatment with AOS delays the accumulation of abscisic acid (ABA), inhibits the expression of ABA signal transduction-related genes, and suppresses cell wall degradation, effectively preserving fruit quality [18]. Moreover, the combined use of *Meyerozyma guilliermondii* and AOS significantly reduces blue mold disease in pear trees by enhancing antioxidative enzyme activity and other key disease-resistance mechanisms, thereby improving the performance of biocontrol agents [19]. In contrast to inducers that offer only singular modes of action or are hampered by specific drawbacks, the broad spectrum of favorable properties associated with AOS translates to its superior development and application potential. The high-purity AOS produced in our laboratory via enzymatic hydrolysis retains the favorable properties of commercial counterparts while being significantly more cost-effective, thereby establishing a solid foundation for its large-scale application.

*H. thailandica* Lg 3 is an antagonistic yeast exhibiting broad-spectrum antifungal activity (Figure S1) isolated and identified from Fuzhou University for controlling postharvest diseases of the banana. It has good biological activity and broad commercial application prospects. However, because of biocontrol yeast’s limited stress resistance ability, its application has been affected by the environment and pathogenic fungi, which makes its biocontrol effect not fully play. Measures must be taken to improve its biocontrol efficiency. Thus, the objective of this study is to assess the impact of AOS on the biocontrol effectiveness of *H. thailandica* Lg 3 against post-harvest diseases of banana fruits and to further investigate the underlying mechanisms. Based on these preliminary findings, we hypothesized that AOS enhances the biocontrol capability of *H. thailandica* Lg 3 by serving as a nutrient source to modulate its growth rate and by altering its transcriptome to improve stress resistance. We believe that the findings from this study will serve as a catalyst for developing innovative approaches to potentiate the efficacy of antagonistic yeasts.

## 2. Materials and Methods

### 2.1. Strains and Fruits

The biocontrol yeast strain *H. thailandica* Lg 3 was initially isolated from the fermentation liquid of longan fruit by the Microbiology Laboratory of the College of Biological Science and Engineering at the Fuzhou University, Fuzhou. Fresh longan fruits, collected from the campus of Fuzhou University, were first immersed in 20% (*v*/*v*) ethanol (Sinopharm Chemical Reagent Co., Ltd., Shanghai, China) for 10 s. Following this, they were rinsed three times with distilled water to complete the initial surface sterilization. The longan epidermis was excised, chopped, and transferred to 1 mL of sterile water, followed by vortexing to mix. The liquid was serially diluted to concentrations ranging from 10^−1^ to 10^−5^. From each dilution, 100 μL was spread onto NYDA (5 g·L^−1^ yeast extract, 8 g·L^−1^ beef extract, 10 g·L^−1^ glucose, 20 g·L^−1^ agar) plates (Aoboxing Universeen Bio-Tech Co., Ltd., Beijing, China) and incubated at 28 °C for 24 h. Following incubation, colonies exhibiting distinct sizes, morphologies, and colors were selected and streaked onto fresh NYDA plates using a three-zone technique for purification. The plates were incubated again at 28 °C, and this streaking process was repeated several times until pure, isolated colonies were obtained (Figure S2). An in vitro antagonism assay against *Colletotrichum musae* was conducted. A strain demonstrating a distinct inhibition zone was selected, designated as Lg 3. The 26S rDNA D1/D2 region (SEQ ID NO: 1) of the Lg 3 was amplified by PCR using the universal forward primer NL1 (5′-GCATATCAATAAGCGGAGGAAAAG-3′) and the reverse primer NL4 (5′-GGTCCGTGTTTCAAGACGG-3′). The amplification was performed using the following thermal cycling protocol: an initial denaturation at 96 °C for 5 min; followed by 35 cycles of denaturation at 96 °C for 20 s, annealing at 56 °C for 30 s, and extension at 72 °C for 30 s; with a final extension at 72 °C for 10 min. The resulting sequence was presented in the Supplementary Material, and it was compared against the NCBI database, and closely related gene sequences were downloaded. All sequences were imported into MEGA5 software, and a phylogenetic tree was constructed using the Neighbor-Joining method (Figure S3). Based on this analysis, the isolated strain was identified as *Hanseniaspora thailandica*.

*H. thailandica* Lg 3 was activated on NYDA and then cultured in NYDB (NYDA without agar) at 28 °C on a rotary shaker (180 rpm). Cells in the logarithmic phase were collected for subsequent experiments.

The pathogen was initially isolated from the anthracnose spot on banana fruits by the Microbiology Laboratory of the College of Biological Science and Engineering at the Fuzhou University, Fuzhou (Figure S4), designated as N3. The ITS rDNA region of the pathogen was amplified by PCR using the universal forward primer ITS1 (5′-TCCGTAGGTGAACCTGCG-3′) and the reverse primer ITS4 (5′-TCCTCCGCTTATTGATATGC-3′). The amplification was carried out under the following conditions: an initial denaturation at 94 °C for 5 min; followed by 35 cycles of denaturation at 94 °C for 40 s, annealing at 58 °C for 40 s, and extension at 72 °C for 60 s; with a final extension at 72 °C for 10 min. The resulting sequence was presented in the Supplementary Material, and it was compared against the NCBI database, and closely related gene sequences were downloaded. All sequences were imported into MEGA5 software, and a phylogenetic tree was constructed using the Neighbor-Joining method (Figure S5). Based on this analysis, the isolated pathogen N3 was identified as *Colletotrichum musae*.

*C. musae* N3 was cultured on a PDA (37 g·L^−1^ potato glucose agar, 6 g·L^−1^ agar) plate at 28 °C for 7 days. The spores were then washed from the plate with sterile distilled water (SDW), and the resulting suspension was filtered through several layers of sterile gauze to obtain a crude spore suspension. The spore concentration was determined using a hemocytometer and adjusted to a final concentration of 1 × 10^5^ spores·mL^−1^ with SDW.

Tianbao banana fruits were procured from an orchard in Zhangzhou, China, and promptly transported to the laboratory. Bananas of uniform size (18–23 cm in length, 130–165 g in weight), free from mechanical damage or disease, were selected for the experiments. The selected fruits were first rinsed with water to remove surface dust and then surface-disinfected by immersing them sequentially in a 20% (*v*/*v*) ethanol solution for 90 s and a 0.5% (*v*/*v*) NaClO solution (Xilong Scientific Co., Ltd., Guangzhou, China) for 15 s. Finally, the fruits were rinsed thoroughly with sterile water to remove any residual disinfectant and air-dried at room temperature for subsequent use.

### 2.2. Biocontrol Assay of AOS-Induced H. thailandica Lg 3

The AOS was enzymatically prepared according to the method of Yan et al. [20] by the Microbiology Laboratory of the College of Biological Science and Engineering at Fuzhou University, Fuzhou, and had a degree of polymerization (DP) of 2–6.

*H. thailandica* Lg 3 was inoculated at 1.0% (*v*/*v*) (from a cell suspension of 1 × 10^8^ cells·mL^−1^) into NYDB medium supplemented with AOS at final concentrations of 0, 5, 10, 20, and 40 mg/L. The culture was incubated at 28 °C and 180 rpm until the logarithmic phase (6 h). The harvested cells were then washed twice with sterile distilled water (SDW) and resuspended in SDW to an appropriate concentration for subsequent assays.

Banana fruits were sprayed with one of the following treatments: (1) SDW (Control); (2) *H. thailandica* Lg 3 cells (1 × 10^8^ cells·mL^−1^); (3–6) *H. thailandica* Lg 3 cells (1 × 10^8^ cells·mL^−1^) that had been induced with 5, 10, 20, or 40 mg/L AOS, respectively. Three hours later, all fruits were inoculated by spraying with a *C. musae* spore suspension (1 × 10^5^ spores·mL^−1^). After air-drying, all treated fruits were packaged in 0.015 mm polyethylene bags and incubated at 28 °C and 80% relative humidity. The disease incidence (rotting rate) was assessed after 12 days of storage according to the method of Zhao et al. [21]. Each treatment consisted of three biological replicates, with each replicate comprising a group of 50 fruits. The entire experiment was repeated twice independently (i.e., on two separate occasions) to verify the reproducibility of the results. The rotting rate was calculated as follows: Rotting rate (%) = (Number of rotting fruits / Total number of fruits) × 100%.

### 2.3. Determination of Polyphenol Oxidase (PPO), Peroxidase (POD), Chitinase (CHI) and Beta-1,3-Glucanase (GLU) Activity in Banana Fruits

The activities of PPO, POD, CHI, and GLU in banana fruit were determined using a frozen tissue sample (1.0 g) pooled from three bananas, as previously described by Wang et al. [7]. One unit of PPO activity was defined as an increase of 0.01 in the optical density at 420 nm per minute. One unit of POD activity was defined as an increase of 0.01 in the optical density at 470 nm per minute. One unit (U) of CHI activity was defined as the amount of enzyme that produces 1 μmol of N-acetylglucosamine per minute per gram of fresh weight. One unit (U) of GLU activity was defined as the amount of enzyme that produces 1 mg of glucose per minute per gram of fresh weight. All enzyme activities are expressed on a fresh weight basis as U·kg^−1^.

### 2.4. Biofilm Formation of AOS-Induced H. thailandica Lg 3

AOS was supplemented into 100 mL of NYDB medium to achieve final concentrations of 0 (control), 5, 10, 20, and 40 mg/L. Each medium was inoculated with 1 mL of an *H. thailandica* Lg 3 suspension (1 × 10^8^ cells·mL^−1^) and cultured at 28 °C with shaking at 180 rpm until the logarithmic phase (6 h). The cells were then collected, washed twice with PBS (50 mmol·L^−1^, pH 7.2), and resuspended to a final concentration of 1 × 10^7^ cells·mL^−1^. This cell suspension was inoculated into a sterile 96-well polystyrene plate and incubated at 28 °C with shaking at 75 rpm for 24 h to evaluate biofilm formation ability, as described by Cordero et al. [22]. Each treatment consisted of three biological replicates. The entire experiment was repeated twice independently (i.e., on two separate occasions) to verify the reproducibility of the results.

### 2.5. Population Dynamics Analysis of AOS-Induced H. thailandica Lg 3 in Banana Wounds and NYDB Medium

The experiment was conducted according to the method of Dhanasekaran et al. [23]. Banana fruits were prepared for inoculation by creating wounds (6 mm in diameter) at the equator using a sterile punch. Each wound was inoculated with 30 μL of one of the following treatments: (1) SDW (Control); (2) *H. thailandica* Lg 3 cells (1 × 10^8^ cells·mL^−1^); or (3–6) *H. thailandica* Lg 3 cells (1 × 10^8^ cells·mL^−1^) that had been pre-induced with 5, 10, 20, or 40 mg/L AOS, respectively.

All treated fruits were individually packaged and placed in an incubator at 20 °C and 80% relative humidity. The time point immediately after the 2 h incubation following the application of *H. thailandica* Lg 3 was designated as day 0. Every two days, tissue samples from the entire wound area were collected using a sterile punch and placed in 100 mL of sterile physiological saline for homogenization. The homogenate was subjected to gradient dilution, plated onto NYDA plates, and incubated at 28 °C for 48 h. The number of colonies was counted and expressed as Log_10_ CFU per wound. Each treatment consisted of three biological replicates. The entire experiment was repeated twice independently (i.e., on two separate occasions) to verify the reproducibility of the results.

AOS was supplemented to 100 mL of NYDB medium to obtain final concentrations of 0 mg/L (control), 5 mg/L, 10 mg/L, 20 mg/L, and 40 mg/L. Then, 1 mL of an *H. thailandica* Lg 3 cell suspension (1 × 10^8^ cells·mL^−1^) was inoculated into the medium and cultured at 28 °C on a rotary shaker at 180 rpm. The growth of the *H. thailandica* Lg 3 culture was monitored by measuring the OD_600_ every 2 h.

### 2.6. Determination of Intracellular Reactive Oxygen Species (ROS), MDA Content and Survival Rate of H. thailandica Lg 3 Under Oxidative Stress

Intracellular ROS levels were measured according to the method of Liu et al. [24]. *H. thailandica* Lg 3 cells, harvested after induction with different concentrations of AOS, were treated with 30 mmol·L^−1^ H_2_O_2_ for 90 min and then washed with PBS buffer. The cells were subsequently resuspended in 25 μmol·L^−1^ DCHF-DA and incubated in the dark for 60 min. After being washed twice with PBS, the cells were observed under a fluorescence microscope, and images were captured.

The MDA content was determined as described by Godana et al. [25]. After the induction culture, *H. thailandica* Lg 3 cells were collected and ground in liquid nitrogen. The protein content was determined using a BCA assay kit (Nanjing Jiancheng Bioengineering Institute, Nanjing, China), and the MDA content was expressed as nmol·mg^−1^ protein.

The survival rate was determined according to the method of Han et al. [26]. *H. thailandica* Lg 3 cells, induced with different concentrations of AOS, were treated with 30 mmol·L^−1^ H_2_O_2_ (Xilong Scientific Co., Ltd., Guangzhou, China) for 90 min. After treatment, the cells were collected and adjusted with PBS to a final concentration of 1 × 10^7^ cells·mL^−1^. Then, 50 μL of the cell suspension was spread onto NYDA plates and incubated for 48 h for colony counting. The survival rate was calculated using the following formula:

Survival rate (%) = (Number of colonies after H_2_O_2_ treatment/Number of colonies before H_2_O_2_ treatment) × 100%.

Each treatment consisted of three biological replicates. The entire experiment was repeated twice independently (i.e., on two separate occasions) to verify the reproducibility of the results.

### 2.7. Determination of the Enzyme Activities of CAT, SOD and GPX

Logarithmic phase *H. thailandica* Lg 3 cells (either AOS-induced or uninduced) were treated with 30 mmol·L^−1^ H_2_O_2_ for 90 min and then processed as described in Section 2.2. The activities of CAT, SOD and GPX in the resulting cell pellets were measured using specific commercial assay kits (Nanjing Jiancheng Bioengineering Institute, Nanjing, China) according to the manufacturers’ protocols.

### 2.8. Inhibition of Spore Germination and Growth In Vitro by AOS-Induced H. thailandica Lg 3

The spore germination rate was determined as described by Zhang et al. [27]. Briefly, *H. thailandica* Lg 3 cell suspension (AOS-induced or uninduced) was added to 50 mL of PDB medium, followed by the addition of 1 mL of a *C. musae* spore suspension (1.0 × 10^5^ spores·mL^−1^). The co-culture was then incubated at 28 °C with shaking at 180 rpm. After 12 h, the spore germination rate was assessed under a microscope. For each treatment, at least 100 spores were observed. A spore was considered germinated when the germ tube length exceeded the spore width. The control treatment consisted of adding an equal volume of SDW in place of the *H. thailandica* Lg 3 suspension. Each treatment consisted of three biological replicates. The entire experiment was repeated twice independently (i.e., on two separate occasions) to verify the reproducibility of the results.

The inhibitory effect on *C. musae* growth was assessed as described by Zhimo et al. [28]. Cell suspensions of *H. thailandica* Lg 3 (AOS-induced or uninduced) were prepared at a concentration of 1 × 10^8^ cells·mL^−1^, with sterile water serving as the control. A well was created in a PDA plate using a sterile cork borer. Then, 30 μL of the *H. thailandica* Lg 3 suspension (1 × 10^8^ cells·mL^−1^) was added to the well. The control group received an equal volume of sterile water. After 3 h, 30 μL of a *C. musae* spore suspension (1 × 10^5^ spores·mL^−1^) was added to the same well. The plates were then incubated at 28 °C for 48 h. The colony diameter of *C. musae* was measured for each of the three replicate plates per treatment. The entire experiment was repeated twice.

### 2.9. Transcriptomics Analysis of AOS-Induced H. thailandica Lg 3

Activated *H. thailandica* Lg 3 was inoculated into NYDB containing 10 mg/L AOS at an inoculum size of 1.0% and cultured for 20 h. Cells were then collected by centrifugation, washed with sterile water, and divided into two groups: AOS-induced and uninduced. Each group consisted of three biological replicates. All samples were immediately frozen in liquid nitrogen to prevent RNA degradation and submitted to Allwegene Technology Co., Ltd. (Beijing, China) for transcriptome sequencing.

### 2.10. RT-qPCR Verification

RT-qPCR was used to validate the expression levels of randomly selected differentially expressed genes (DEGs). Total RNA, extracted from *H. thailandica* Lg 3 samples, was first reverse-transcribed into cDNA using the PrimeScript™ RT reagent kit (TransGen Biotech, Beijing, China) with random primers. The resulting cDNA was then used as a template for RT-qPCR analysis. RT-qPCR was performed with three technical replicates for each gene using cDNA from three independent biological replicates. The selected genes and their corresponding specific primers are listed in Table S1.

### 2.11. Statistical Analysis

All data were analyzed by analysis of variance (ANOVA), using Origin 8.0 and IBM SPSS Statistics 25.0. And the data were expressed as mean ± SE (*n* ≥ 3). The statistical significance was determined at *p* < 0.05. Statistical significance in the figure is indicated by letter notation. The letter display follows the standard method for indicating significant differences: means are arranged in descending order, with the highest mean labeled ‘a’. This mean is then compared sequentially with lower means, assigning the same letter to those not significantly different. When a significant difference is encountered, the new mean is labeled with the next consecutive letter, and the comparison process continues iteratively until all means are appropriately marked. Means sharing any common letter do not differ significantly at α = 0.05.

## 3. Results

### 3.1. Effect of AOS on Biocontrol Efficiency of H. thailandica Lg 3 Against C. musae on Banana Fruits

**Figure 1:**
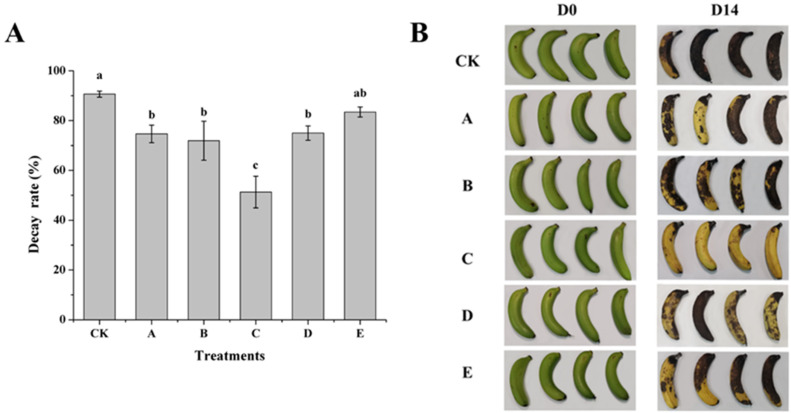
(**A**) Effect of *H. thailandica* Lg 3 spraying treatment with different concentrations of AOS-induced culture on the rotting rate of banana fruits. (**B**) Effect of *H. thailandica* Lg 3 spraying treatment with different concentrations of AOS-induced culture on the biocontrol efficacy of banana fruits. CK: sterile water; A: Lg 3; B: Lg 3 induced by 5 mg/L AOS; C: Lg 3 induced by 10 mg/L AOS; D: Lg 3 induced by 20 mg/L AOS; E: Lg 3 induced by 40 mg/L AOS. The description of the letter notation method used to indicate statistical significance in the figures can be found in Section 2.11.

The biocontrol efficacy of *H. thailandica* Lg 3 against banana fruit rot was significantly enhanced by induction with AOS (Figure 1). While all tested concentrations of AOS improved performance compared to the uninduced *H. thailandica* Lg 3, the induction with 10 mg/L AOS was most effective, reducing the rot rate by approximately 40% after 14 days of storage—more than double the reduction achieved by other concentrations (Figure 1A,B). This indicates that AOS induction, particularly at an optimal concentration, can profoundly boost the bio-control activity of *H. thailandica* Lg 3.

### 3.2. Effect of H. thailandica Lg 3 Enhanced with AOS on POD, PPO, CHI and GLU Activities in Banana Fruits

**Figure 2:**
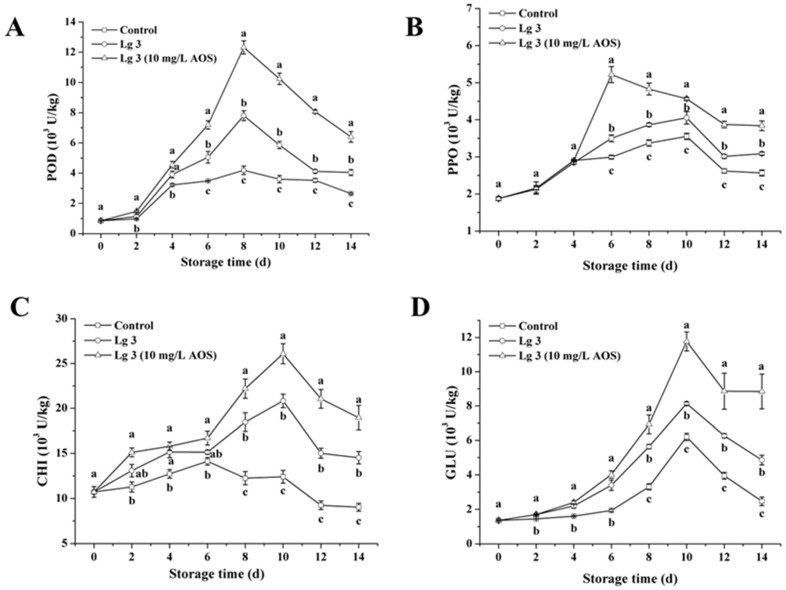
Effect of *H. thailandica* Lg 3 enhanced with AOS on POD, PPO, CHI and GLU activities in banana fruits stored for 14 d at 28 °C. (**A**): POD; (**B**): PPO; (**C**): CHI; (**D**): GLU. The description of the letter notation method used to indicate statistical significance in the figures can be found in Section 2.11.

Our results demonstrate that AOS-induced *H. thailandica* Lg 3 potentiates the fruit’s innate defense by orchestrating a superior enzymatic response (Figure 2). Compared to the yeast alone, the AOS-induced yeast (10 mg/L) elicited a more rapid induction of PPO (enhancing early lignification) and, most notably, sustained high activity of the pathogenesis-related (PR) proteins CHI and GLU. The sustained activity of these chitinolytic and glucanolytic enzymes implies continuous degradation of the pathogen’s structural components, providing a durable biochemical barrier against infection [17]. This enhanced and prolonged defense signature explains the significant reduction in disease symptoms achieved with AOS-induced Lg 3, as visually documented in Figure 1.

### 3.3. Effect of AOS on Biofilm Formation of H. thailandica Lg 3

**Figure 3:**
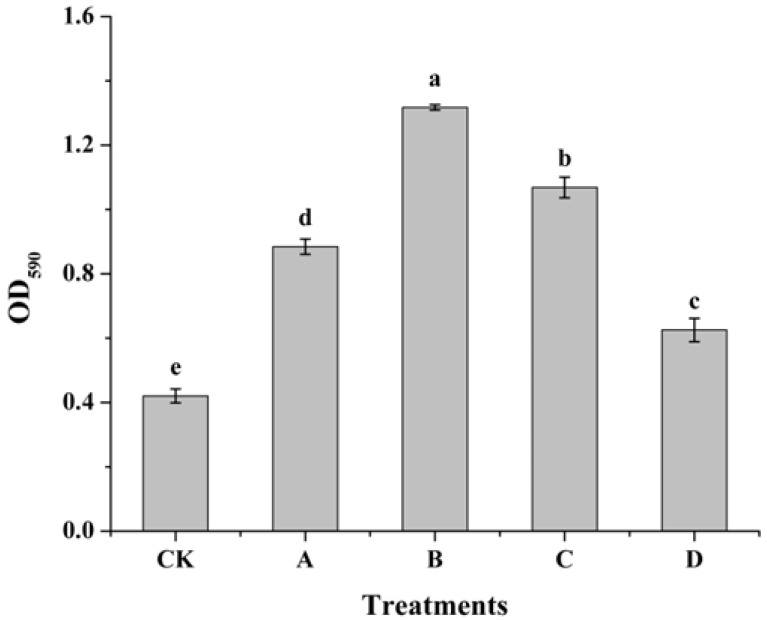
Effect of different concentrations of AOS on biofilm formation capacity of *H. thailandica* Lg 3. CK: Lg 3; A: Lg 3 induced by 5 mg/L AOS; B: Lg 3 induced by 10 mg/L AOS; C: Lg 3 induced by 20 mg/L AOS; D: Lg 3 induced by 40 mg/L AOS. The description of the letter notation method used to indicate statistical significance in the figures can be found in Section 2.11.

Induction with AOS significantly enhanced the biofilm-forming capacity of *H. thailandica* Lg 3 in a concentration-dependent manner, with 10 mg/L identified as the optimal concentration (Figure 3). The biofilm density (OD_590_) of yeast induced with 10 mg/L AOS was 1.31, representing a substantial (over three-fold) increase over the non-induced control. This robust enhancement in biofilm formation suggests an improved ability of the yeast to adhere to surfaces, a trait critical for successful colonization and biocontrol.

### 3.4. Effect of AOS on Population Dynamics of H. thailandica Lg 3 in NYDB and Banana Wound

**Figure 4:**
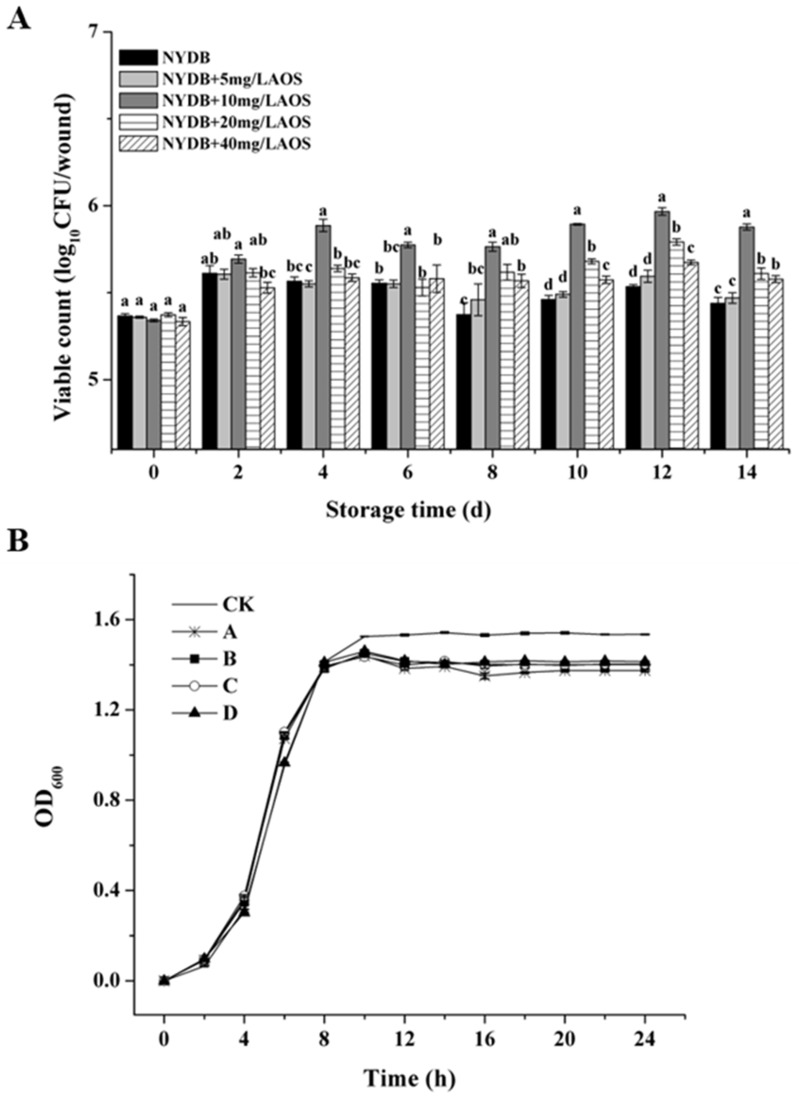
Effect of different concentrations of AOS on the colonization ability and growth dynamics of *H. thailandica* Lg 3. (**A**): colonization ability; (**B**): growth dynamics, CK: Lg 3; A: Lg 3 induced by 5 mg/L AOS; B: Lg 3 induced by 10 mg/L AOS; C: Lg 3 induced by 20 mg/L AOS; D: Lg 3 induced by 40 mg/L AOS. The description of the letter notation method used to indicate statistical significance in the figures can be found in Section 2.11.

Our results clarify a crucial aspect of biocontrol enhancement: AOS priming optimizes *H. thailandica* Lg 3 for persistence in the target environment, not for unrestricted growth. Figure 4 demonstrates that AOS-induced Lg 3, particularly at 10 mg/L, achieved markedly superior colonization on banana wounds compared to the uninduced yeast (Figure 4A), which correlates perfectly with its biocontrol efficacy. This occurred despite AOS showing an inhibitory effect in nutrient-rich NYDB (Figure 4B). This clear dichotomy indicates that AOS reprograms the yeast’s physiology, likely shifting its priority from proliferation to stress resilience and competitive fitness. This adaptation is key to maintaining a robust, protective population in the nutrient-scarce and competitive fruit wound niche, thereby ensuring effective biocontrol.

### 3.5. Effects of AOS on ROS, MDA Content and Survival Rate of H. thailandica Lg 3 Under Oxidative Stress

**Figure 5:**
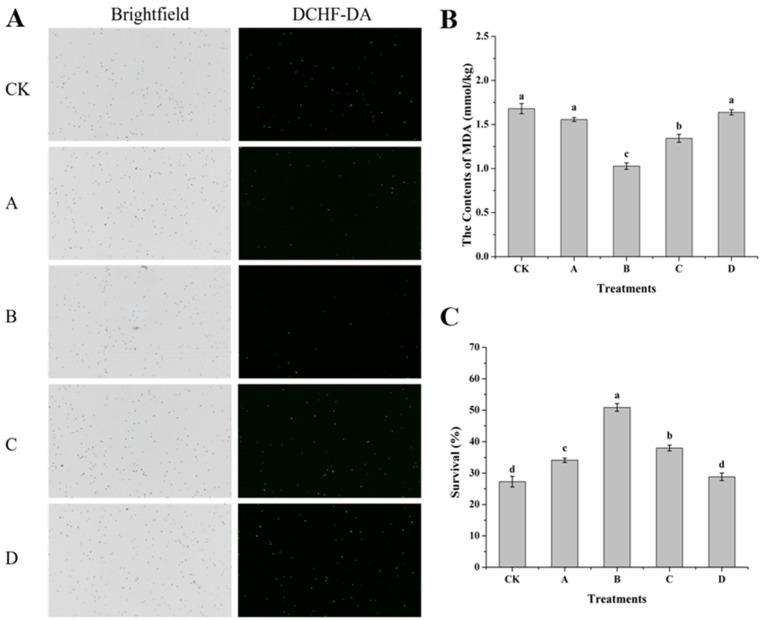
Effect of different concentrations of AOS on the ROS, MDA and survival rate of *H. thailandica* Lg 3. (**A**): ROS; (**B**): The contents of MDA; (**C**): Survival. CK: Lg 3; A: Lg 3 induced by 5 mg/L AOS; B: Lg 3 induced by 10 mg/L AOS; C: Lg 3 induced by 20 mg/L AOS; D: Lg 3 induced by 40 mg/L AOS. The description of the letter notation method used to indicate statistical significance in the figures can be found in Section 2.11.

Induction with AOS, particularly at 10 mg/L, conferred a profound enhancement of oxidative stress tolerance in *H. thailandica* Lg 3 (Figure 5). This protective effect was demonstrated by a substantial reduction in intracellular ROS (Figure 5A) and a concomitant decrease in membrane lipid peroxidation (as measured by MDA content, Figure 5B). Critically, this mitigation of oxidative damage directly resulted in a near-doubling of cell viability (50.87% vs. 27.29% in the control, Figure 5C). We conclude that AOS priming does not merely bolster general cellular defenses, but specifically and efficiently preserves membrane integrity under stress. This maintained integrity is essential for survival and directly explains the yeast’s improved fitness in the oxidative environment of a fruit wound, thereby ensuring robust biocontrol activity.

### 3.6. Effect of AOS on the Activity of Antioxidant Enzymes and AOS Content of H. thailandica Lg 3

**Figure 6:**
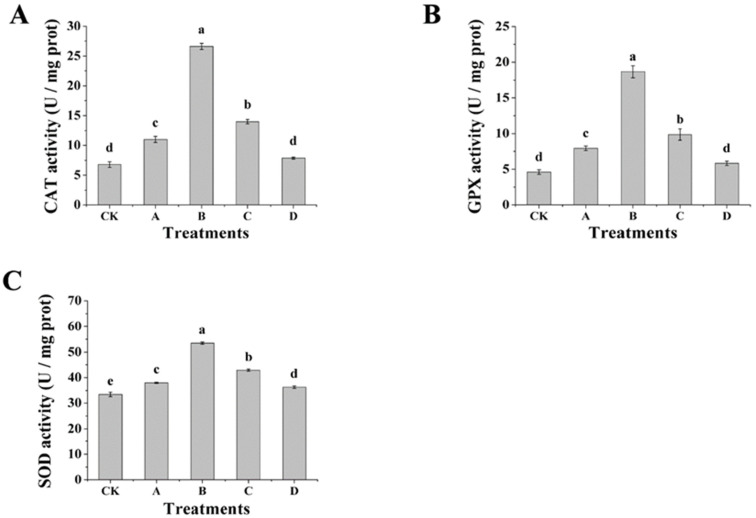
Effect of different concentrations of AOS on the antioxidant ability of *H. thailandica* Lg 3 (**A**): CAT; (**B**): GPX; (**C**): SOD. CK: Lg 3; A: Lg 3 induced by 5 mg/L AOS; B: Lg 3 induced by 10 mg/L AOS; C: Lg 3 induced by 20 mg/L AOS; D: Lg 3 induced by 40 mg/L AOS. The description of the letter notation method used to indicate statistical significance in the figures can be found in Section 2.11.

The enhanced oxidative stress tolerance in AOS-induced *H. thailandica* Lg 3 (Figure 5) is mechanistically grounded in a coordinated upregulation of its core enzymatic antioxidant system (Figure 6A–C). Pre-induction with AOS, particularly at 10 mg/L, significantly boosted the activities of catalase (CAT), superoxide dismutase (SOD), and glutathione peroxidase (GPX). Critically, these enzymes function in a synergistic cascade: SOD first converts superoxide radicals into hydrogen peroxide, which is then efficiently detoxified by the elevated activities of both CAT and GPX [11]. This pre-configured, multi-level defense network explains the significantly reduced ROS burden (Figure 5A) and the consequent near-doubling of cell survival (Figure 5C). Thus, AOS priming does not merely increase enzyme activity but equips the yeast with a rapid-response capability to neutralize oxidative bursts, a key determinant for its fitness in the stressful plant environment.

### 3.7. Effect of AOS on Inhibition of Spore Germination and Growth of H. thailandica Lg 3

**Figure 7:**
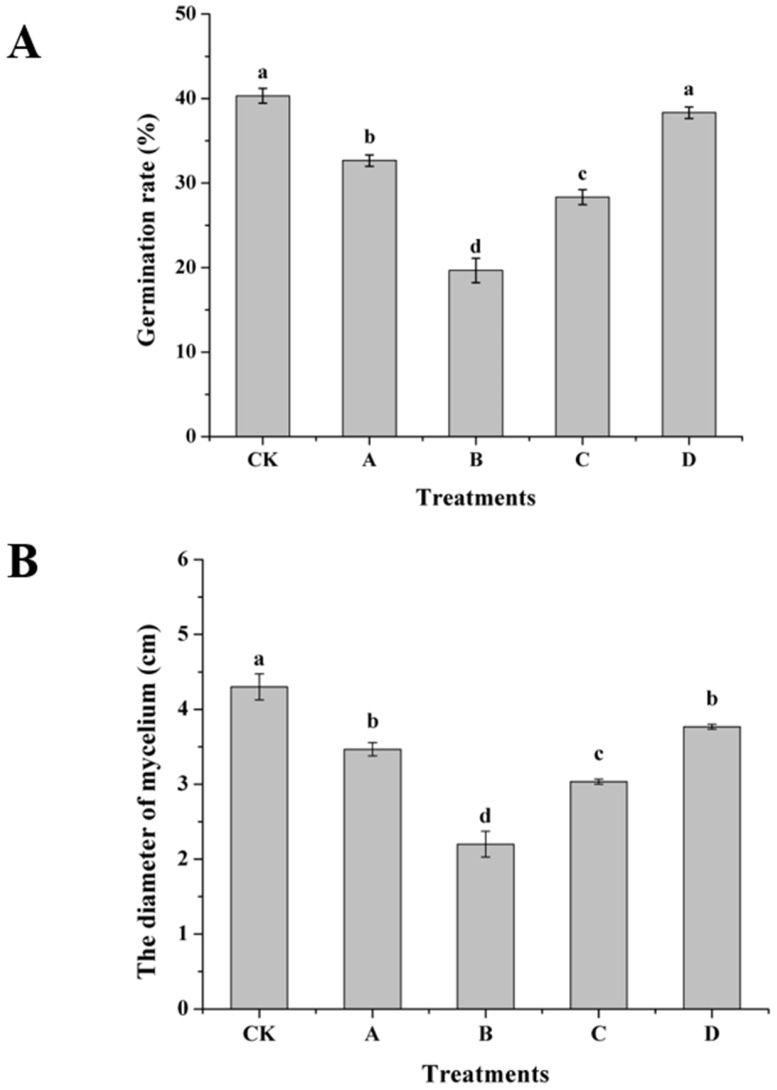
Effect of different concentrations of AOS on inhibition of *C. musae* by *H. thailandica* Lg 3 (**A**): Germination rate; (**B**): The diameter of mycelium. CK: Lg 3; A: Lg 3 induced by 5 mg/L AOS; B: Lg 3 induced by 10 mg/L AOS; C: Lg 3 induced by 20 mg/L AOS; D: Lg 3 induced by 40 mg/L AOS. The description of the letter notation method used to indicate statistical significance in the figures can be found in Section 2.11.

AOS induction drastically amplified the direct antagonism of *H. thailandica* Lg 3 against *C. musae*, effectively suppressing both hyphal growth and spore germination (Figure 7A,B). The optimally induced (10 mg/L) yeast nearly halved the pathogen’s mycelial diameter and spore germination rate compared to the non-induced control. This potent dual-mode inhibition at the pathogen level is the key determinant of the enhanced biocontrol performance observed in vivo.

### 3.8. RNA-Seq Analysis

The transcriptomic data for *H. thailandica* Lg 3, both before and after AOS treatment, were provided by Allwegene Technology Co., Ltd. (Beijing, China). A summary of the sequencing statistics is presented in Table 1. All samples exhibited a Q30 score above 93% and a GC content within the range of 35–40%, indicating high-quality sequencing. Therefore, the data are reliable and suitable for subsequent transcriptomic analysis.

**Figure 8:**
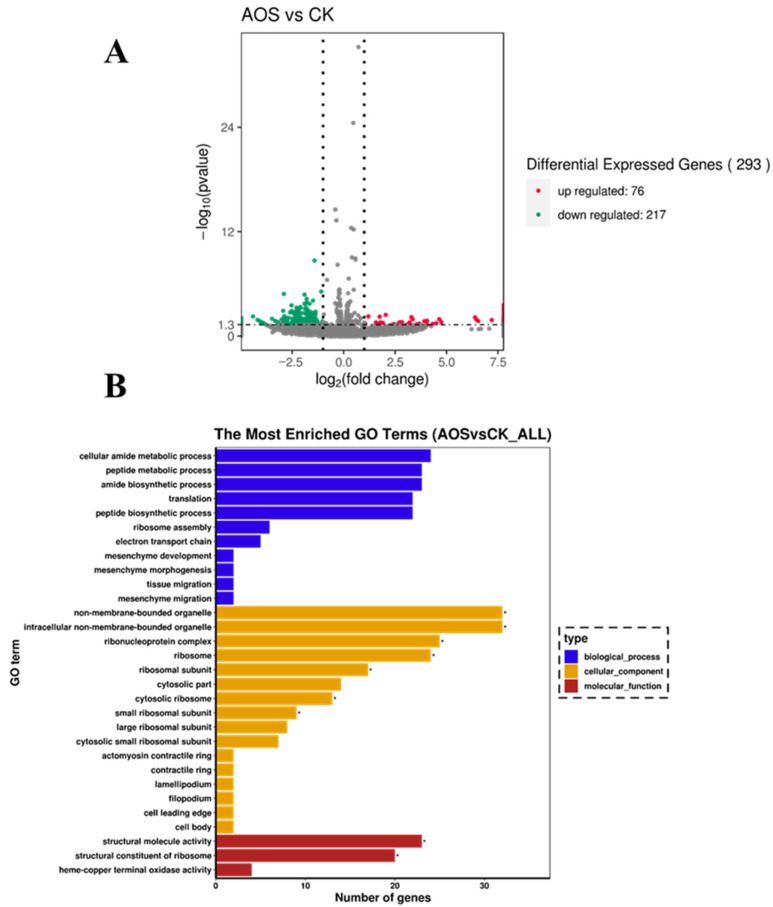
(**A**). Volcanic map of differentially expressed genes. The figure shows the differentially expressed genes of strain *H. thailandica* Lg 3 before and after the 10 mg/L AOS-induced culture. Each point in the differential expression volcanic map represents a gene, and the abscissa represents the logarithmic value of the multiple of the differential expression of a gene in two samples; Longitudinal coordinates represent the negative logarithmic value of the error detection rate. Red dots represent genes with up-regulation, green dots represent genes with down-regulation, and blue dots represent genes with no significant difference in expression. (**B**) Go enrichment histogram.

Transcriptome analysis of AOS-induced *H. thailandica* Lg 3 revealed a significant shift in its genetic program, with 293 differentially expressed genes (Figure 8A). The predominance of downregulated genes (217 vs. 76 upregulated) indicates that AOS priming triggers a comprehensive reallocation of cellular resources, which underpins the observed enhancements in stress tolerance and biocontrol efficacy.

### 3.9. GO Enrichment

GO enrichment analysis indicated that the AOS-induced transcriptional shift in *H. thailandica* Lg 3 is fundamentally linked to processes critical for its biocontrol efficacy (Figure 8B). The strong enrichment for ribosome biogenesis and translation aligns with the need for rapid protein synthesis to support stress tolerance and biofilm development. Simultaneously, changes in terminal oxidase activity suggest an adjustment in energy metabolism, and the enrichment of cytoskeletal components hints at improved adhesion capabilities. This coordinated response at the molecular level effectively primes the yeast for the challenges of the phyllosphere environment.

### 3.10. KEGG Enrichment

**Figure 9:**
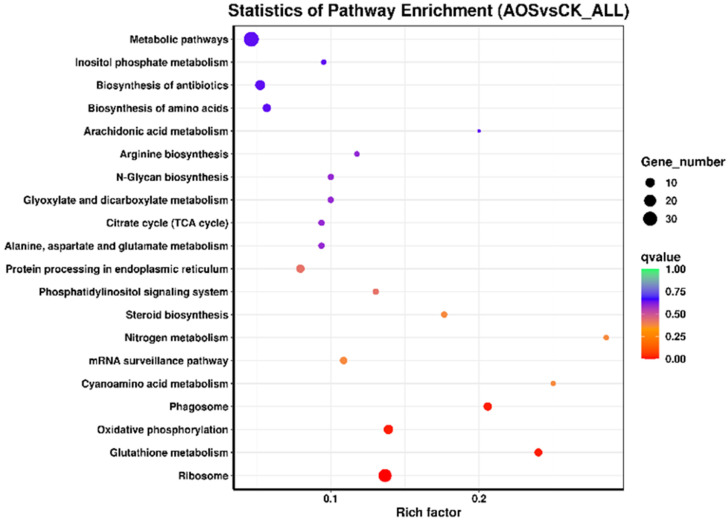
Scatter plot of KEGG enrichment of differentially expressed genes. Longitudinal coordinates represents the name of the pathway, and the abscissa represents the Rich factor. The size of the dots indicates the number of differentially expressed genes in this pathway, and the color of the dots corresponds to different q-value ranges.

KEGG pathway enrichment analysis can help us further understand the physiological and metabolic pathways involved by different genes and the metabolic pathway of AOS regulating *H. thailandica* Lg 3. A cumulative total of 205 DEGs were enriched in 52 metabolic pathways, with the top 20 enriched pathways depicted in Figure 9. Among them, the top 10 pathways with the most significant enrichment were ribosome, glutathione metabolism, oxidative phosphorylation, phagosome, steroid biosynthesis, nitrogen metabolism, mRNA surveillance pathway, cyanobacterial amino acid metabolism, protein processing in endoplasmic reticulum, and phosphatidylinositol signaling system. These pathways mainly involved yeast’s genetic information and cellular and metabolism processes.

### 3.11. RT-qPCR Validation

Table 2 shows a high correlation between the results of RT-qPCR and RNA-seq data analyses. These data indicate that the outcomes of transcriptome sequencing are reasonably accurate and reliable.

## 4. Discussion

To overcome the environmental instability that often hinders biocontrol yeasts [5], we explored AOS as a priming agent for *H. thailandica* Lg 3. Our findings confirm that AOS induction is a viable strategy to enhance biocontrol consistency, as it synergistically improves both the ecological fitness of the yeast—a phenomenon observed in other antagonistic yeasts [29]—and the defense capacity of the fruit host, likely through a process akin to microbe-induced resistance (MIR) that primes plant defenses. This coordinated enhancement underscores the value of host–microbe co-management in developing next-generation biocontrol solutions.

For biocontrol yeasts to succeed in practice, they must withstand the host’s defensive oxidative burst [30]. However, in addition to inhibiting the infection of pathogenic fungi, the ROS will threaten the survival rate of biocontrol yeast, thus affecting the biocontrol efficiency of biocontrol yeast [31]. Our work confirms that the oxidative stress tolerance of *H. thailandica* Lg 3 is not a fixed trait but can be decisively enhanced through AOS induction. This “priming” effect is a pivotal factor in its improved performance, as it allows the yeast to better colonize the wound site under oxidative pressure [10]. Therefore, augmenting stress tolerance through elicitors like AOS is a crucial strategy for developing more reliable and effective biocontrol formulations.

The ability to form biofilms and effectively colonize plant surfaces is widely recognized as a critical determinant of biocontrol success, as it enables the protective microbial agent to establish a persistent presence at the infection site [8,23]. Our results indicate that AOS induction directly enhances this key competency in *H. thailandica* Lg 3. The resultant improvement in its establishment and persistence on banana fruit wounds underpins the *H. thailandica* Lg 3’s increased spatial and nutritional competitiveness, ultimately translating to the significantly stronger biocontrol performance observed in our study.

The enhanced oxidative stress tolerance in AOS-induced *H. thailandica* Lg 3 is also mechanistically linked to the potentiated activity of its core antioxidant enzyme system (SOD, CAT, GPX), which are known to function synergistically to mitigate oxidative damage [32]. This coordinated upregulation at the optimal 10 mg/L concentration provided a critical line of defense, effectively reducing ROS accumulation and subsequent membrane damage (lipid peroxidation). This safeguarding of cellular integrity is fundamental to maintaining viability under duress [33], thereby ensuring a robust population of the biocontrol agent can be sustained in the challenging fruit wound environment to exert its protective effect. Similarly, Wang et al. [34] found that glycine (1 mM) induced higher SOD and CAT activities in *Sporidiobolus pararoseus*, which mitigated its oxidative stress and concurrently activated host defense enzymes in apple. Despite differences in the species and inducing agents involved, these cases collectively indicate that enhancing the antioxidant capacity of antagonistic yeast represents a key mechanism through which inducers can improve biocontrol efficacy.

The efficacy of a biocontrol agent often hinges on its direct inhibitory capacity against the pathogen [35]. Our findings confirm that AOS induction significantly amplifies this key trait in *H. thailandica* Lg 3, strengthening its ability to impede both the germination and vegetative growth of *C. musae*. This potentiated direct antagonism, consistent with the effects of other elicitors like methyl jasmonate [36], is an integral component of the overall enhancement in decay control, working in concert with improved colonization and stress tolerance to deliver superior biocontrol outcomes.

The statistical results of the DEGs showed that the *H. thailandica* Lg 3 induced by 10 mg/L AOS were 293 DEGs compared with the uninduced group, including 76 up-regulated genes and 217 down-regulated genes. KEGG pathway enrichment showed that the top 3 physiological metabolic pathway involved in the DEGs was the ribosomal metabolism pathway, glutathione metabolism pathway, and oxidative phosphorylation pathway.

In the ribosome pathway, 25 differentially expressed genes were annotated, among which 4 genes were up-regulated after AOS induction, and 21 genes were downregulated. The ribosome is an essential cellular organelle responsible for protein synthesis and requires much energy. Research has shown that cells can adapt to changes in growth conditions by reducing ribosomal protein synthesis [37]. Under oxidative stress, cells may actively downregulate the expression of ribosomal protein genes to conserve energy and reduce growth rate [38,39]. When exogenous proteins are overexpressed in *Saccharomyces cerevisiae*, the expression of 30 ribosomal protein genes is downregulated, and at the same time, the yeast growth rate significantly decreases [40]. RPS3 is a 40S ribosomal protein involved in protein synthesis and participates in DNA repair, transfer, and apoptosis [41]. Transcriptomic results showed that after induction with 10 mg/L AOS treatment, the *S3e* gene in the ribosome was significantly downregulated, leading to reduced synthesis of RPS3 and energy savings in yeast cells to improve survival under adverse conditions [42]. RPS0A is a structural component of the 40S ribosome involved in cytoplasmic translation and ribosomal biogenesis and is associated with cell growth rate [43]. After induction with 10 mg/L AOS treatment, the *SAe* gene in the ribosome was significantly downregulated, leading to decreased biogenesis of RPS0A. It reduced cell growth to enhance protein expression efficiency in response to oxidative stress. RPL10 is a structural component of the 60S ribosome and is a highly conserved ribosomal protein with functions in biogenesis and protein synthesis. After induction with 10 mg/L AOS, the *L0e* gene in the ribosome was significantly downregulated, leading to decreased synthesis of RPL10, reduced energy consumption, and preparation of yeast cells to combat oxidative stress [44].

Regarding energy metabolism, the oxidative phosphorylation pathway is the primary pathway for cellular energy production during aerobic cell growth, generating a significant amount of adenosine triphosphate (ATP) to provide energy support for yeast cells [45]. Complex IV is the cytochrome c oxidase complex that transfers electrons from cytochrome c to molecular oxygen [46]. The genes encoding cytochrome c oxidase subunit 1 (*COX1*), cytochrome c oxidase subunit 2 (*COX2*), and cytochrome c oxidase subunit 3 (*COX3*), are three subunits of cytochrome c oxidase, which play essential roles in catalyzing and regulating cellular activities and have significant regulatory effects on the accumulation of complexes I, III, IV, and V in the oxidative phosphorylation pathway [47]. Transcriptomic results showed that after induction with 10 mg/L AOS treatment, the expression of genes *COX1*, *COX2*, and *COX3* was up-regulated in the oxidative phosphorylation pathway. This phenomenon may be due to the induction of yeast-related gene expression by AOS, leading to an increase in the relative content of cytochrome c oxidase within yeast cells, promoting the accumulation of complexes I, III, IV, and V, enhancing yeast cell’s ability to synthesize energy and improving vitality. This finding is consistent with that of Song et al. [48]. Sulfuric acid is an essential coenzyme in mitochondrial metabolism that can regulate the enzyme activity of the 2-ket acid dehydrogenase complex. Acyl-carrier protein gene (*ACP1*) is believed to be associated with the biogenesis of thioctic acid (a precursor of sulfuric acid) [49]. The results showed a trend towards up-regulating the *ACP1* gene after induction with 10 mg/L AOS treatment, which may promote the synthesis of sulfuric acid in yeast cells, providing more energy for yeast cells. Hence, the induced *H. thailandica* Lg 3 can maintain vitality under oxidative stress. *Cytb*, encoded by the cytochrome b gene, participates in electron transfer in mitochondria and is part of mitochondrial respiratory complex III [50]. After induction with 10 mg/L AOS treatment, *Cytb* is up-regulated, accelerating electron transfer and synthesis of complex III in mitochondria, accelerating energy synthesis in yeast cells, and enhancing yeast’s resistance to stress.

In the glutathione metabolism pathway, GSH (glutathione) is involved in cellular defense against oxidative stress, detoxification of exogenous substances, regulation of cell apoptosis and proliferation, amino acid transport and other functions [51]. The biosynthesis of glutathione involves two steps, which are catalyzed by two distinct enzymes. The initial step is catalyzed by γ-glutamylcysteine synthetase, which synthesizes γ-glutamylcysteine from L-glutamate and cysteine [52]. The transcriptional activity of the γ-glutamylcysteine synthetase gene (*GSH1*) is intricately linked to GSH synthesis. In the glutathione metabolism pathway, the expression of the *GSH1* gene was up-regulated in *H. thailandica* Lg 3 induced by 10 mg/L AOS, leading to an increase in GSH content in yeast cells, which helps to remove reactive oxygen species and improves yeast cell survival. Upon exposure to reactive oxygen species, yeast cells activate glutathione peroxidase (*GPx*), which plays an essential role in the yeast’s defense against oxidative stress [53]. The transcriptomic results showed that the expression of the *GPx* gene was up-regulated in *H. thailandica* Lg 3 induced by 10 mg/L AOS, leading to an increase in *GPx* content in yeast cells. *GPx* can utilize glutathione as an electron donor to catalyze the reduction of hydrogen peroxide, thereby reducing intracellular hydrogen peroxide levels and alleviating oxidative damage [54]. Gamma-glutamyltranspeptidase facilitates the transfer of the gamma-glutamyl group from GSH and other gamma-glutamyl-containing compounds to amino acids and peptides. [55]. Research has shown that when gamma-glutamyltranspeptidase levels are low, cellular GSH is inert [56]. The transcriptomic results showed that the gene encoding gamma-glutamyltranspeptidase (*ECM38*) was up-regulated in *H. thailandica* Lg 3 induced by 10 mg/L AOS, promoting the antioxidant function of GSH and enhancing the antioxidant capacity of yeast cells.

**Figure 10:**
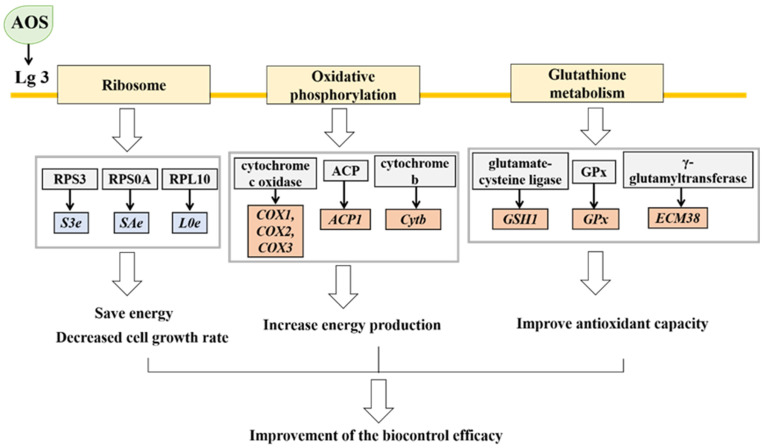
Effect of adding 10 mg/L AOS induction on genes on the *H. thailandica* Lg 3 ribosomal pathway. The blue box represents downregulation, and the orange box represents the regulation level not changed.

Under environmental stress, cells moderate growth by downregulating ribosomal gene expression while enhancing translational efficiency to maintain essential protein synthesis [57]. As shown in Figure 10, after induction with 10 mg/mL AOS, the expression of ribosome-related genes in *H. thailandica* Lg 3 was downregulated. This may be due to the slight stress caused by AOS as an inducer of yeast growth. The downregulation of ribosome-related gene expression in *H. thailandica* Lg 3 leads to a decrease in cell growth rate, saving energy to cope with stress, thereby improving the survival rate of *H. thailandica* Lg 3 under oxidative stress conditions and ultimately enhancing its biocontrol efficiency. At the same time, the genes related to oxidative phosphorylation pathway in *H. thailandica* Lg 3 were upregulated, resulting in the production of more energy in yeast cells and maintaining higher vitality. Therefore, the induced *H. thailandica* Lg 3 exhibits stronger resistance under oxidative stress. The upregulation of genes related to glutathione metabolism pathway in *H. thailandica* Lg 3 increases the content of GSH in yeast cells, which helps to remove reactive oxygen species in cells and improve yeast survival rate. This is consistent with our previous experimental results.

## 5. Conclusions

Our experimental data indicates that AOS can enhance the ability of *H. thailandica* Lg 3 to inhibit *C. musae*, suggesting it as an effective strategy to improve the biocontrol efficacy of *H. thailandica* Lg 3. The enhancement of *H. thailandica* Lg 3’s biocontrol efficiency may be positively correlated with its ability to form biofilms, the ability of colonization, oxidative stress tolerance, antioxidant enzymes and AOS content. In addition, transcriptome results showed that 10 mg/L AOS can down-regulate the related ribosome genes of *H. thailandica* Lg 3, improve the efficiency of protein synthesis, and enhance the survival ability under oxidative stress conditions. In conclusion, 10 mg/L AOS improved the stress resistance and the nutrient competition ability of *H. thailandica* Lg 3, thus improving the biocontrol effect. The outcomes of this study lay the groundwork for future research on the application of biocontrol yeasts for the management of postharvest diseases in fruits and vegetables.

## Figures and Tables

**Table 1:** Quality assessment of sample sequencing data.

Sample Name	Clean Reads	Clean Bases	Q30 (%)	GC Content (%)
CK_1	41,776,868	6.31 G	93.69%	39.04%
CK_2	37,422,730	5.65 G	93.87%	39.19%
CK_3	42,329,510	6.39 G	93.79%	38.81%
AOS_1	42,189,562	6.37 G	94.02%	38.78%
AOS_2	41,830,758	6.32 G	93.65%	39.01%
AOS_3	40,589,372	6.13 G	94.09%	38.98%

**Table 2:** Results of relevant genes verified by transcriptomic sequencing with RT-qPCR.

Gene	RNA-Seq (Log2FC)	RT-qPCR(Log2FC)
L-lactate dehydrogenase	−1.8812	−1.8600 ± 0.0043
beta-1 tubulin	−1.8262	−1.7100 ± 0.0030
ribosomal protein P0	−1.3298	−1.2033 ± 0.0014
40S ribosomal protein S6-B	−2.0875	−2.2433 ± 0.0018
60S ribosomal protein L8	−2.0982	−2.5733 ± 0.0026
60S ribosomal protein L16	−1.5576	−1.5000 ± 0.0043
60S ribosomal protein L15	−1.6818	−1.6967 ± 0.0008
60S ribosomal protein L10	−1.4456	−1.5367 ± 0.0058
glutamate–cysteine ligase	6.4516	5.8167 ± 0.0004
glutathione peroxidase	7.1974	7.3033 ± 0.0029

## Data Availability

The raw data supporting the conclusions of this article will be made available by the authors on request.

## Supplementary Materials

The following supporting information can be downloaded at: https://www.mdpi.com/article/10.3390/jof11120824/s1, Table S1: Primers used for real-time PCR analysis. Figure S1: Broad-spectrum antifungal activity of Lg-3. (A, B) *Alternaria* sp., imaged at day 8; (C, D) *Botryosphaeria dothidea*, day 5; (E, F) *Gloeosporium musarum*, day 5; (G, H) *Colletotrichum acutatum*, day 4; (I, J) *C. gloeosporioides*, day 4; (K, L) *Fusarium* sp., day 4; (M, N) *Neofusicoccum parvum*, day 3. Figure S2: The strain morphology of Lg3. Figure S3: Molecular genetic identification results of Lg-3. Figure S4: The strain morphology of N3. Figure S5: Molecular genetic identification results of N3.
