# Supplementary material for: Improvement of Biocontrol Efficiency of Hanseniaspora thailandica Induced by Alginate Oligosaccharide Against Banana Anthracnose Caused by Colletotrichum musae
Source: J Fungi (Basel). 2025 Nov 21;11(12):824. doi: 10.3390/jof11120824 (PMC12734200; doi:10.3390/jof11120824)
Supplement: Supplementary file 1 [file jof-11-00824-s001.zip › jof-3920927-supplementary.pdf]

Table S1. Primers used for real-time PCR analysis.

Gene ID	Gene description	Forward primers (5'-3')	Reverse primers (3'-5')
TRINITY_ DN1218_c0_g1	L-lactate dehydrogenase	ATGGTCAAAGTCGC AATTCTTGGCG	TCATAGCTTGGAAG AGTCTAGGATGAAACTC TT
TRINITY_ DN1336_c1_g2	beta-1 tubulin	ATGAGAGAAATCAT TCATATCTCGACAGGTC AG	TTATTCAAAATTCTC AGTGATTGGTTCATCTTG GTT
TRINITY_ DN2105_c0_g1	actin	ATGGATTCTGGTATG TTCTAGCGCTT	TTAGAAACACTTGT GGTGAACGATAGATGGA
TRINITY_ DN1567_c0_g1	ribosomal protein P0	ATGGGAGGCATTCG TGAAAAGAAAG	TTAATCGAATAAAC CGAAACCCATGTCGT
TRINITY_ DN15273_c0_g1	40S ribosomal protein S6-B	ATGAAGGTATGATTT ATGATACTATTATTGAGG GGCAA	TTAAGCCTTCAAAG AAGAAGCTCTTCTCTTT C
TRINITY_ DN643_c0_g1	60S ribosomal protein L8	ATGGGTATGTTTTTA TTATATCGCATAATTATG GCAAATG	TTAATCTTGGGTCTT TTGAGAACCACGT
TRINITY_ DN781_c0_g1	60S ribosomal protein L16	ATGTCTGTTGAACC AGTTGTTGTCATTG	TTAGTAACCCAAAG CGGCTAATTGTTTAGC
TRINITY_ DN1321_c0_g1	60S ribosomal protein L15	ATGGGAGCCTACAA ATATTTAGAAGAATTAGA AAGAAA	TCATTTTCTATACCT CCATAACGACAGAGTAT TTTGT
TRINITY_ DN846_c0_g1	60S ribosomal protein L10	ATGGCTAGAAGACC AGCTAGATGTTACA	TTAAGCTTGAGCAG CAAAGTATTCTGG
TRINITY_ DN2860_c0_g1	glutamate-c ysteine ligase	ATGGGACTCTTAGC TTTGGGCAC	TTAACATTTGCTTTC TATTGAAGGCTTATTTTT TTTTACATATTC

TRINITY_ DN1589_c0_g1	glutathione peroxidase	ATGACCACATCTTTT TATGATTTAGAATGCAAG GA	TCATTTACTTAACAG GCTTTGGATTTCTTGGT
TRINITY_ DN6307_c0_g1	acyl carrier protein 2, mitochondrial	ATGTTTAGATCCGTT TGCCGCATTT	TTAGTTTGCGTCGG GATTGGAAGC
TRINITY_ DN1886_c0_g1	cytochrome c oxidase subunit I	ATGGTACAAAGATG ATTATATTCAACAAATGC AAAAG	TTAAGATTGTACAG CTGGTGTATTAAATGAGT GTA

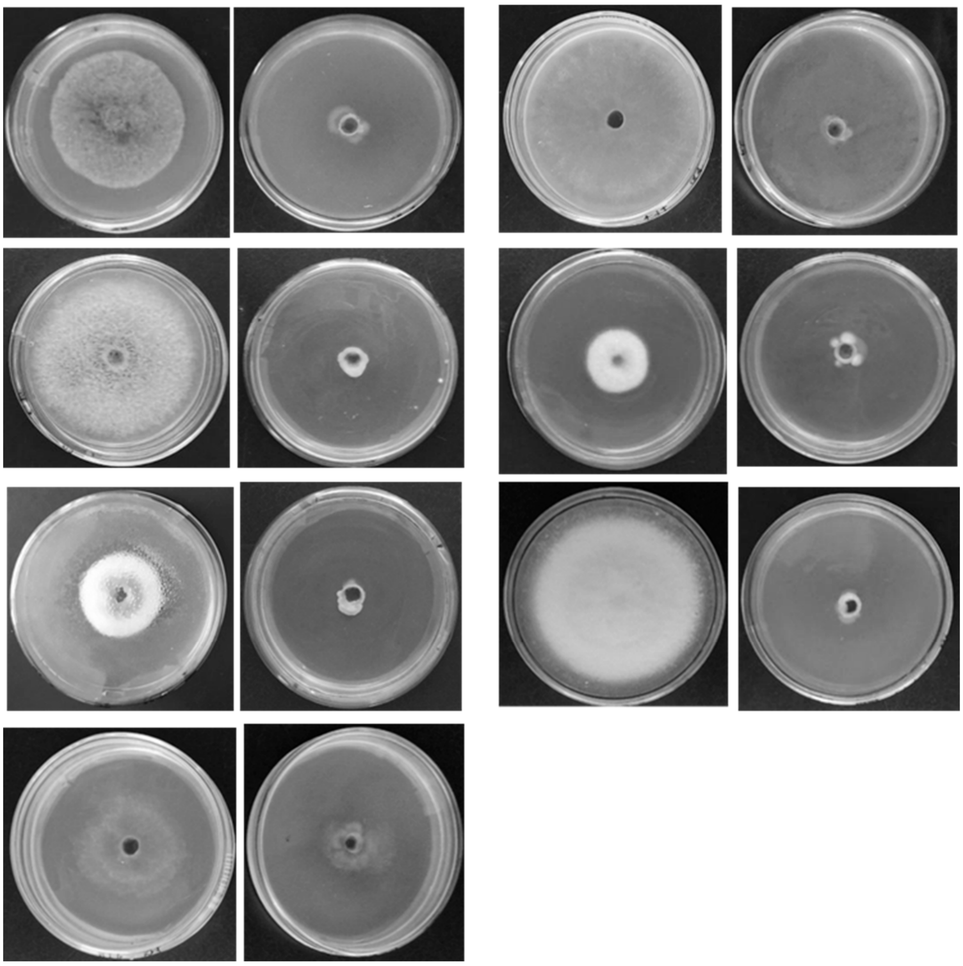

Figure S1. Broad-spectrum antifungal activity of Lg-3. (A, B) *Alternaria sp*., imaged at day 8; (C, D) *Botryosphaeria dothidea*, day 5; (E, F) *Gloeosporium musarum*, day 5; (G, H) *Colletotrichum acutatum*, day 4; (I, J) *C. gloeosporioides*, day 4; (K, L) *Fusarium sp.*, day 4; (M, N) *Neofusicoccum parvum*, day 3.

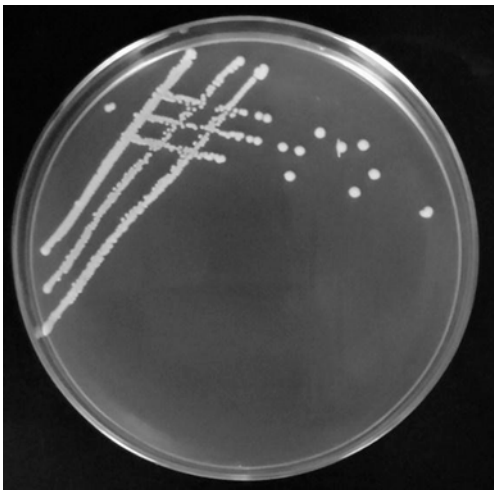

Figure S2. The strain morphology of Lg3.

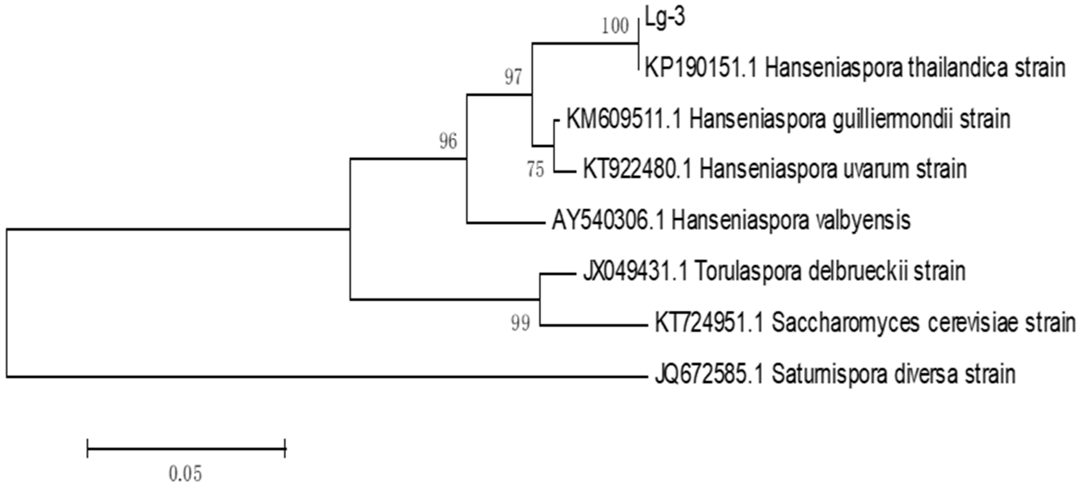

Lg-3 26S rDNA D1/D2 region, SEQ ID NO.1：

5'-CCAAGGGGATACTTAGTACGGCGAGTGAGCGGTAAAAGCTCAAATTTGA
AATCTGGTACTTTCAGTGCCCGAGTTGTAATTTGTAGAATTTGTCTTTGATTA
GGTCCTTGTCTATGTTCCTTGGAACAGGACGTCATAGAGGGTGAGAATCCC
GTTTGGCGAGGATACCTTTTCTCTGTAAGACTTTTTCGAAGAGTCGAGTTG
TTTGGGAATGCAGCTCAAAGTGGGTGGTAAATTCCATCTAAAGCTAAATATT
GGCGAGAGACCGATAGCGAACAAGTACAGTGATGGAAAGATGAAAAGAA
CTTTGAAAAGAGAGTGAAAAAGTACGTGAAATTGTTGAAAGGGAAGGGCA
TTTGATCAGACATGGTGTTTTTTTAATGTGCAAGTCTCTCGTGGACATGTGC
CTCTGGAAATTAACACTGGGCCAACATCAGTTCTGGCGGCAGGATAAATCA
TTAAGAATGTAGCTACCTCGGTAGTGTTATAGCTTTTTGGAATACTGCCAGC
TGGGATTGAGGACTGCGCTTCGGCAAGGATGTTGGCATAATGGTTAAATGC
CGCCCGTCTTGAAACCACGGACCA-3'

Figure S3. Molecular genetic identification results of Lg-3.

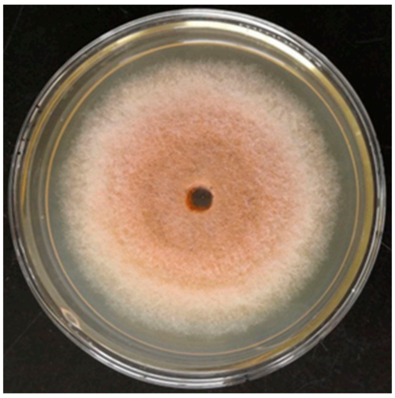

Figure S4. The strain morphology of N3.

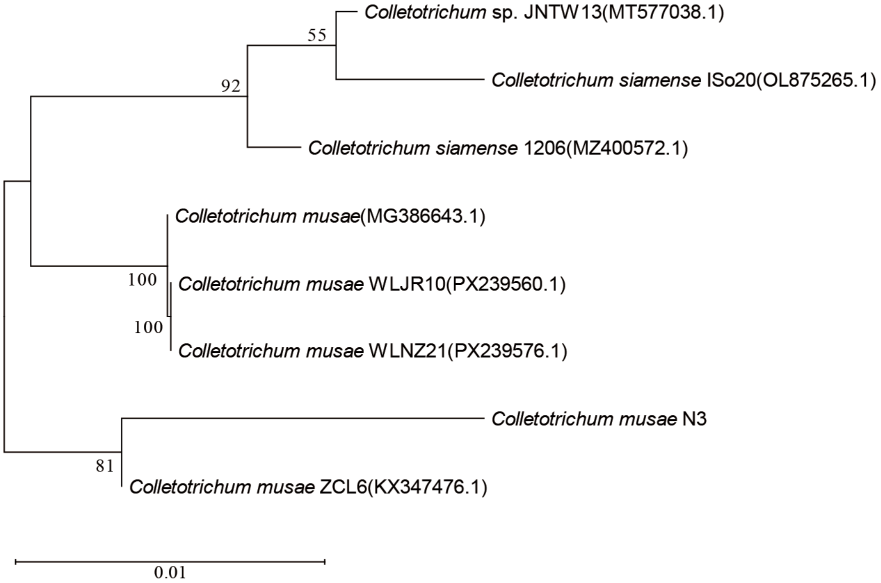

N3 ITS rDNA region：

5'-TGGGGGATCGGGAGTTACGCTCTGCACCCTTTGTGACATACCTATAACTG
TTGCTTCGGCGGGTAGGGTCCCCGTGACCCTCCCGGCCCCCCGCCCCCGGG
CGGGTCGGCGCCCGCCGGAGGATAACCAAACTCTGATTTAACGACGTTTCT
TCTGAGTGGTACAAGCAAATAATCAAAACTTTTAACAACGGATCTCTTGGT
TCTGGCATCGATGAAGAACGCAGCGAAATGCGATAAGTAATGTGAATTGCA
GAATTCAGTGAATCATCGAATCTTTGAACGCACATTGCGCCCGCCAGCATT
CTGGCGGGCATGCCTGTTCGAGCGTCATTTCAACCCTCAAGCTCTGCTTGG
TGTTGGGGCCCTACAGCAGATGTAGGCCCTCAAAGGTAGTGGCGGACCCTC
CCGGAGCCTCCTTTGCGTAGTAACTTTACGTCTCGCACTGGGATCCGGAGG
GACTCTTGCCGTAAAACCCCCCAATTTTCCAAAGGTTGACCTCGGATCAGG
TAGGAATACCCGCTGAACTTAAGCATATCAAAAGGCCGGAGGAA-3'

Figure S5. Molecular genetic identification results of N3.
